# Ashwagandha: Is It Safe? Part 2: A Preclinical Evidence Review

**DOI:** 10.1002/ptr.70090

**Published:** 2025-09-18

**Authors:** Elizabeth M. Williamson, Thomas Brendler

**Affiliations:** ^1^ School of Pharmacy, University of Reading Reading UK; ^2^ Department of Botany and Plant Biotechnology University of Johannesburg Johannesburg South Africa; ^3^ Department of Pharmaceutical Sciences Pharmacognosy Institute, Retzky College of Pharmacy, University of Illinois Chicago Chicago Illinois USA; ^4^ National Center for Naturopathic Medicine, Southern Cross University East Lismore Australia

**Keywords:** adaptogen, ashwagandha, preclinical investigations, safety, *Withania*

## Abstract

The preclinical evidence for the safety of ashwagandha ( *Withania somnifera* (L.) Dunal, AS) is reviewed, and its preparations (extracts) and constituents, from the general toxicity in animal models to in vitro and cell culture studies, which may elucidate mechanisms of action and explain clinical case reports. Most studies and reviews conclude that AS is a remarkably safe herb, but cases of liver toxicity, mainly reversible cholestasis or severe jaundice and pruritus, have been reported without being predicted by preclinical evidence. Further work is needed to clarify the constituents responsible and patient‐related issues surrounding them. Several constituents, such as withaferin‐A, have demonstrable antitumorigenic effects in animal models of liver cancer and injury induced by ischemia, cytotoxic drugs, and radiotherapy. AS has not shown genotoxicity or mutagenicity in standard tests; on the contrary, it was protective against chromosome abnormalities or micronuclei formation induced by known clastogenic agents. A folkloric reputation for inducing abortion or sterility is not borne out of preclinical experiments, and rare reports of cardiotoxicity could not be substantiated.

## 1 Introduction

Ashwagandha ( *Withania somnifera* (L.) Dunal, AS) is widely used in traditional medicine, especially throughout Asia, where all parts of the plant are used and often combined with other herbs. There is little evidence of either safety or toxicity for these mixtures. Phytomedicines containing extracts of root, and to a lesser extent leaf, manufactured according to GMP standards, are increasingly popular in the West for indications related to their effects on the central nervous system: insomnia, stress, anxiety, and as neuroprotectants in cases of cognitive decline, stroke, and other degenerative conditions. Speers and colleagues have comprehensively reviewed the evidence for the neuropsychiatric effects of AS and its constituents, and mechanisms of action which may explain them, but suggest that “the fact that many of the standardized, active extracts contain 70%–95% of uncharacterized materials highlights the possibility of additional, unidentified active compounds” (Speers et al. 2021). This has implications for the safety of different types of extracts.

Literature reviews of AS generally conclude that it is safe and well‐tolerated (Bashir et al. 2023; Paul et al. 2021; Yadav et al. 2024), but safety issues have been raised by regulators (Brendler et al. 2026). These include clinical case reports of hepatotoxicity (Björnsson et al. 2020; Gnecco et al. 2022; Ireland et al. 2021; Lubarska et al. 2023; Philips et al. 2023; Rattu et al. 2022), undesirable endocrinological effects (Fry et al. 2022; Gannon et al. 2014; Hayashi et al. 2024; Van Der Hooft et al. 2005; Ziganshina and Joseph 2022), and purported abortifacient activity (Brendler 2026). The clinical evidence will be reviewed in more detail elsewhere (Brendler and Williamson 2025), but preclinical and mechanistic studies are discussed here to provide context.

The therapeutic effects of AS are largely ascribed to a range of withanolides, which are steroidal lactones based on the ergostane skeleton. AS contains > 40 withanolides, although > 1200, of varying structural types, have been discovered distributed throughout the Solanaceae. The main constituents of AS root are listed in Table 1, and constituents unique to AS leaves are listed in Table 2. Withanolides A and D, withaferin A and withanone, are cited as the most significant pharmacologically (Paul et al. 2021; Shinde et al. 2023; Zhang et al. 2024).

**TABLE 1:** Constituents identified in *Withania somnifera* root.

Chemical class	Individual compounds and classes	References
Withanolides (steroidal lactones)/glycosides	Withanolides A, E–L, Y, withaferin‐A, withanone, withasomniferols A–C, withasomniferin A, withasilolides A–F, withasomnidienone, witharistatin, physagulin D, coagulin H, Q, S withastramonolide, witha‐2,24‐dienolide, plus many derivatives/withanosides I–VII	Bashir et al. (2023), Dar et al. (2015), Kim et al. (2019), Matsuda et al. (2001), Paul et al. (2021), Shinde et al. (2023)
Alkaloids	Anagyrine, anahygrine, anaferine ashwagandhine, ashwagandhinine, cuscohydrine, somninine, somniferine, somniferinine, solasidine, tropine, pseudotropine, dl‐isopelletierine, withasomnine, visamine, withanine, withaninine, pseudowithaninine, withanmine	Bashir et al. (2023), Paul et al. (2021), Shinde et al. (2023)
Phytosterols/glycosides	ß‐Sitosterol, stigmasterol, stigmastadiene, cholesterol, diosgenin/sitoindosides VII–X	Bashir et al. (2023), Paul et al. (2021)
Phenylpropanoid esters	Withaninsams A and B, N‐trans‐feruloyl methoxytyramine, N‐trans‐feruloyltyramine, acetosyringone	Baek et al. (2019)
Polyphenols	Chlorogenic acid, catechin, scopoletin, kaempferol, naringenin, quercetin	Bashir et al. (2023), Paul et al. (2021)

**TABLE 2:** Constituents identified in *Withania somnifera* leaf.

Chemical class	Individual compounds and classes	References
Withanolides (steroidal lactones)/glycosides	Withanolides A–T, Z, withanosides VI–XI, coagulin Q, withaferin A, witharistatin	Shinde et al. (2023)
Alkaloids	Anaferine (bis (2‐piperidylmethyl) ketone), tropine, isopelletierine, 3α‐tigloyloxtropine, cuscohygrine, 3‐tropyltigloate, hygrine, dl‐isopelletierine, withasomnine, mesoanaferine, withanine, somniferine, hentriacontane, withananine, visamine, ashwagandhine, and pseudowithanine	Kirson and Gottlieb (1980), Saleem et al. (2020)

Other parts of the plant, such as the fruits, contain many further constituents, and the phytochemistry continues to be investigated. The leaves contain withanolides M–T, flavonoids, and numerous unconfirmed compounds (Shinde et al. 2023). Alkaloids are present in all parts of the plant, with the leaves usually having a higher concentration than the roots. Very low levels of toxic alkaloids such as yohimbine (~0.00007%) and strychnine (~0.00001%) in the dry herb (plant parts not stated) have been reported (Filipiak‐Szok et al. 2017) but are not recorded by other authors (Shinde et al. 2023). Notably, the fruits have been shown to contain a series of alkaloids, withanamides A–I, which have been found to protect cells from β‐amyloid, a protein implicated in Alzheimer's disease (Jayaprakasam et al. 2010).

## 2 Materials and Methods

A comprehensive search of standard scientific databases (PubMed, Scopus, and Google Scholar) was conducted up to March 2025 using combinations of the following keywords: ashwagandha, *W. somnifera* , constituents, withanolide, preclinical, safety, toxicity, cardiotoxicity, hepatotoxicity, and endocrine toxicity. Additional studies found in references were also included. While the primary focus was on preparations derived from the AS root, studies using other parts of the plant were also considered where appropriate. However, these are not definitive as the literature contains numerous compounds where the plant parts were not stated in the original references but subsequently ascribed to the root. Further sources of confusion are the toxic withanolides (e.g., withangulatins), withaphysalins, and physalins found in species of *Physalis* (Damu et al. 2007). These have diverse biological activities, including abortifacient effects (Mohana et al. 1979), but have not been reported in *W. somnifera* .

## 3 Results and Discussion

Numerous studies have been conducted on the various extracts and constituents of AS. These are summarized in Table 3 below. For assessment of potential toxicity, studies in which the constituents were not analyzed or standardized have been included, providing that the plant material was authenticated.

**TABLE 3:** Preclinical toxicity tests of *Withania somnifera* extracts and constituents.

Species/cell type	Extract or compound	Dose(s), route	Test type duration	Observations and results	References
Rat	Root total alkaloidal extract	Various i/p single	Acute toxicity	LD_50_ 465 mg/kg	Malhotra et al. (1965)
Mouse				LD_50_ 432 mg/kg	
Rat	Root alcoholic extract	Various p/o single	Acute toxicity	LD_50_ > 1000 mg/kg	Bhakuni et al. (1969)
Rat	Crude alkaloidal extract	Approx. 1 g extract p/o daily, in feed	Subacute toxicity, 14 days	Centrilobular degeneration of liver; vascular congestion in lung, and tubular degeneration in kidney	Arseculeratne et al. (1985)
Mouse	Root hydro‐alcoholic extract	Various i/p single doses	Acute toxicity	NOAEL > 1100 mg/kg, LD_50_ 1260 mg/kg	Sharada et al. (1993)
Rat		100 mg/kg i/p daily	Subacute toxicity, 30 days	Reduction in weight of spleen, thymus, and adrenal glands in males. Blood acid phosphatase increased in both sexes. No other adverse effects observed.	
Rat	Root 10% hydro‐alcoholic extract	2000 mg/kg p/o single	Acute toxicity	No mortality or overt toxic effects. NOAEL > 2000 mg/kg	Prabu et al. (2013)
		500–2000 mg/kg p/o daily	Subacute toxicity, 28 days	No effect on organ or body weight, histopathology, hematological parameters NOAEL > 2000 mg/kg	
Pregnant rat	Root 10% hydro‐alcoholic extract	500–2000 mg/kg p/o daily on Days 5–19 gestation	Prenatal maternal and fetal toxicity	No maternal or fetal toxicity, no changes in body weight of parent females, number of corpora lutea, implantations, viable fetuses, external, skeletal, or visceral malformations	Prabu and Panchapakesan (2015)
Rat	Root extract standardized to ≥ 3% withaferin	2000 mg/kg p/o single	Acute toxicity	No mortality or signs of toxicity. NOAEL > 2000 mg/kg	(S. B. Patel et al. 2016)
		500–2000 mg/kg p/o daily	Subacute toxicity, 28 days	No effect on organ or body weight, histopathology, hematological parameters NOAEL > 2000 mg/kg	
Human PBL	Root extract	35 μg/mL, 70 μg/mL	Genotoxicity induced by H_2_O_2_	Protection against DNA damage induced by H_2_O_2_: reduction in sister chromatid exchange in human peripheral blood lymphocyte assay	N. Kumar et al. (2016)
Rat	Root extract standardized to 35% withanolides	2000 mg/kg p/o single	Acute toxicity	No mortality or signs of toxicity. NOAEL > 2000 mg/kg	Antony et al. (2018)
		100, 500, and 1000 mg/kg p/o daily	Subchronic toxicity, 90 days	Hematology/biochemistry profiles, histopathology of major organs of control and treated animals unchanged. NOAEL > 1000 mg/kg bw.	
Rat	Whole plant aqueous‐methanolic extract	100, 300, and 1000 mg/kg p/o daily	Subacute toxicity 28 days	No change in body and organ weights, histopathology, hematology, biochemical parameters, ophthalmology, urinalysis, and so on.	Balkrishna et al. (2022)
Rat	Root prepared with cow ghee and milk (0.4% withaferin A)	2000 mg/kg p/o single	Acute toxicity	No neurological toxicity, mortality or gross pathological organ changes	Gurav et al. (2023)
Rat	Root aqueous extract	200, 400, and 800 mg/kg p/o daily	Subacute toxicity 28 days	No changes in hematocrit, hemoglobin, erythrocytes, platelets, blood clotting time, serum ALT, AST, or ALP levels, or histopathology.	Langade et al. (2023)
RAW 264.7 cells	Root extracts, WNL, and Alk‐enriched	0.005–0.1 mg/mL	MTT cell viability	Lower toxicity observed in extracts enriched with alkaloids (ALK) compared to unenriched extracts and withanolide (WNL)‐enriched extracts	Orrù et al. (2023)
Rat	Root aqueous extract standardized to > 5% withanolides; containing < 0.1% withaferin A.	500, 1000, and 2000 mg/kg p/o daily	Subchronic toxicity, 90 days	Hematological and biochemical parameters, organ weights, and histopathology unchanged. NOAEL > 2000 mg/kg.	Kalaivani et al. (2023)
Mouse		2000 mg/kg p/o single	Acute toxicity	No mortality or signs of toxicity within 3 days. NOAEL > 2000 mg/kg.	Kalaivani et al. (2024)
Mouse		500, 1000, and 2000 mg/kg p/o daily	Micronucleus test	No increase in incidence of micronuclei in erythrocytes ≤ 2000 mg/kg body weight	
Ames		0.156–5 mg	Genotoxicity	No effect on reverse mutation up to 5 mg/plate	
Human PBL		0.25–2 mg/mL	Clastogenesis	No chromosome aberrations in human lymphocytes at doses ≤ 2 mg/ml	
Calf DNA	Withanone	200 μM	DNA‐GSH adduct	Withanone forms non‐labile adducts with DNA and deoxy nucleosides, in competition with glutathione (GSH)	Siddiqui et al. (2021)
Mouse	Withaferin‐A	2000 mg/kg p/o single	Acute toxicity	NOAEL > 2000 mg/kg	Gupta et al. (2022)
		10, 70, and 500 mg/kg p/o daily	Subacute toxicity 28 days	No change in serum chemistry, hematology, and histopathology at any dose levels, NOAEL > 500 mg/kg.	
Rat	Root aqueous extract standardized to > 2.5 w/w% total withanolides	2000 mg/kg p/o single	Acute toxicity	LD_50_ > 2000 mg/kg	Wangikar et al. (2024)
		250, 500, and 1000 mg/kg p/o daily	Subchronic toxicity, 90 days	NOAEL > 1000 mg/kg	

### 3.1 General Toxicity Studies

Most of the rodent toxicity studies report that root extracts are generally safe, giving a NOAEL (No Observed Adverse Effect Level) in rodents of 2000 mg/kg body weight when administered orally. This equates to a human equivalent of approximately 22 g daily, which is much greater than extract doses recommended.

#### 3.1.1 Toxicological Investigations of Branded Extracts

An acute oral toxicity study in Sprague–Dawley rats (OECD‐423) with Shoden (Arjuna Natural) demonstrated safety at dosages of up to 2000 mg/kg body weight, with an LD_50_ exceeding 2000 mg/kg body weight. Furthermore, a 90‐day repeated dose toxicity study in rats provided further evidence of the absence of toxic effects when administered at 1000 mg/kg body weight (Antony et al. 2018).

An acute oral toxicity study in Wistar rats (OECD‐423) with a standardized root extract, KSM‐66 (Ixoreal) showed safety at dosages of up to 2000 mg/kg body weight (Kalaivani et al. 2024). The study further evaluated the mutagenic potential in the bacterial reverse mutation (Ames II) at concentrations of 0.156–5.00 mg/plate, chromosomal aberration at concentrations of 0.25–2.00 mg/mL, and micronucleus test at up to 2000 mg/kg body weight in Swiss albino mice. A 28‐day subacute toxicity study in Wistar rats (OECD‐407) with KSM‐66 at doses of 200, 400, and 800 mg/kg body weight did not cause any pathological effects on any of the observed parameters (hematological, biochemical, gross necropsy and histopathology) (Langade et al. 2023). A 90‐day repeated dose toxicity study in Wistar rats (OECD‐408) with KSM‐66 at doses of up to 2000 mg/kg body weight did not exhibit any clinical signs of toxicity (weight, feeding, hematology, biochemistry, thyroid hormone levels, histopathology, morbidity or mortality), setting the NOAEL for KSM‐66 at 2000 mg/kg body weight per day (Kalaivani et al. 2023).

Acute and subacute toxicity studies in Wistar rats (OECD‐407 and 408) with root extract AgeVel/Witholytin (Verdure Sciences) were conducted with doses up to 2000 mg/kg body weight. No relevant findings (clinical, ophthalmic, body weight, feed, serum chemistry, hematology, and histopathology) were reported (S. B. Patel et al. 2016).

Acute and subchronic (90 days) toxicity studies in Sprague–Dawley rats (OECD‐407 and 408) were performed with LongeFera (PhytoVeda). Safety was demonstrated for up to 2000 mg/kg bw, and no NOAEL could be established for doses up to 1000 mg/kg bw (Wangikar et al. 2024).

#### 3.1.2 Acute Studies

Those generally show little toxicity for orally administered Ashwagandha extracts in both acute and subacute tests. Single dose tests in rats (Sprague–Dawley or Wistar) demonstrated safety at dosages of up to 2000 mg/kg body weight (Antony et al. 2018; Gurav et al. 2023; S. B. Patel et al. 2016; Prabu and Panchapakesan 2015; Prabu et al. 2013). Another group found a similar lack of toxicity in different models with Wistar rats and Swiss mice at the same dose (Kalaivani et al. 2024).

A traditional preparation (AS Ghrita, made of roots, cow ghee, and milk, 0.4% withaferin A) was found safe at up to 2000 mg/kg body weight in a single‐dose acute toxicity study in Sprague–Dawley rats (OECD‐425) (Gurav et al. 2023).

An early study administered a hydro‐alcoholic extract of the root to Swiss albino mice by intraperitoneal (i/p) injection and reported a NOAEL > 1100 mg/kg and the LD_50_ at 1260 mg/kg, but as AS extracts are always taken orally, the relevance of this study is unclear (Sharada et al. 1993).

#### 3.1.3 Repeated Dose Studies

Subacute studies in rats have also shown a lack of toxicity. Twenty‐eight‐day studies of extract KSM‐66 did not elicit any pathological effects on hematological and biochemical parameters, gross necropsy, and histopathology at doses up to 800 mg/kg body weight, the highest dose tested (Langade et al. 2023). Ninety‐day studies were equally non‐toxic at a dose of 1000 mg/kg daily, and up to 2000 mg/kg (Kalaivani et al. 2023), neither reported changes in mortality, weight, hematology, biochemistry, thyroid hormone levels, or histopathology. A whole plant (roots, stem, leaves, berries and seeds) aqueous methanolic (1:1) extract showed no toxicologically relevant findings in a 28‐day subacute toxicity study (OECD‐407) in rats at doses up to 1000 mg/kg body weight per day (Balkrishna et al. 2022).

A subacute toxicity study over 30 days reported that 100 mg/kg caused a reduction in the weight of the spleen, thymus, and adrenal in male Wistar rats, and an increase in blood acid phosphatase in both sexes. No other adverse effects were observed, but as with their acute test, the extract was administered by an intraperitoneal injection, and its relevance is questionable (Sharada et al. 1993).

#### 3.1.4 Developmental Toxicity

A 10% hydroalcoholic root extract, given by oral gavage to pregnant Wistar rats on Days 5–19 gestation, produced no maternal toxicity at doses of 500–2000 mg/kg daily, and no fetal toxicity (Prabu and Panchapakesan 2015). There were no changes in body weight of parent females, number of corpora lutea, implantations, viable fetuses, external, skeletal, or visceral malformations. Surprisingly, this is the only modern preclinical study on pregnant animals found in the literature. The dried extract, prepared using 80% methanol, contained 0.043% ± 0.004% withaferin A and was dispersed in water prior to administration. The results confirmed the lack of toxicity of the same extract in nonpregnant animals in both acute and subacute tests (Prabu et al. 2013).

### 3.2 Toxicity Associated With Individual Compounds and Phytochemical Classes

#### 3.2.1 Alkaloids

In contrast to the lack of toxicity of total extracts of AS root and whole plant, alkaloidal extracts of the root show acute toxic effects in both mice and rats, with LD_50_ 465 mg/kg in rats and LD_50_ 432 mg/kg in mice (strains not cited) (Malhotra et al. 1965). In a 14‐day feeding study, Sprague–Dawley rats given a crude alkaloid extract (dose approximately 1 g daily) showed pathohistological changes to various organs: centrilobular degeneration of the liver, vascular congestion of the lung, and tubular degeneration of the kidney (Arseculeratne et al. 1985). The contribution of the alkaloid fraction to the overall toxicity of the root extract is, however, not clear. Orrù et al. (2023), while investigating anti‐inflammatory activity and toxicity of extracts of different chemical composition, observed not only greater anti‐inflammatory effects but also significantly lower toxicity in the MTT cell viability by extracts enriched with alkaloids compared to withanolide‐enriched extracts (Orrù et al. 2023). This may be simply a question of dose and route of administration: in the study by Malhotra et al. (1965) very high doses (> 400 mg/kg) of a mixed alkaloidal extract were administered by intraperitoneal injection, which is not comparable to the alkaloid dose ingested as part of an oral preparation of a total root extract.

#### 3.2.2 Withanolides

Pretorius et al. (2009) compared the cytotoxicity of AS water and methanol extracts and found the methanol extracts to be more toxic to MRC‐5 cells (a human embryonic lung‐derived fibroblast cell line) in the MTT cell viability test (Pretorius et al. 2009). They attributed this to withaferin A and withanolide D being more easily extracted using methanol. However, no adverse effects were reported in BALB/c mice fed isolated withaferin at a single dose of 2000 mg/kg, and no changes in serum chemistry, hematology, and histopathology were reported in a 28‐day subacute toxicity test at levels up to 500 mg/kg (Gupta et al. 2022).

Orrù et al. (2023) showed that very low dosages of withanolide‐enriched extract strongly reduced cell viability in RAW 264.7 cells (a macrophage‐like cell line, originating from leukemia virus‐transformed cells in BALB/c mice), also using the MTT test (Orrù et al. 2023). This test measures cell viability in vitro but may not reflect toxicity in vivo after oral administration, and most animal tests suggest the safety of extracts, even those containing high concentrations of withanolides: for example, root extracts standardized to 35% withanolides did not show toxic effects in rats either at a single dose of 2000 mg/kg nor when fed for 90 days at 1000 mg/kg daily (Antony et al. 2018).

### 3.3 Mutagenicity, Genotoxicity, and Clastogenic Agents

The bacterial reverse mutation test (BRMT, Ames II test) identifies mutagenicity in standard strains of *Salmonella typhimurium* . Kalaivani et al. (2024) used this method to test AS root extracts and found no mutagenic effects at doses up to 5 mg/plate. The study included a chromosome aberration assay using human peripheral blood lymphocytes (PBLs), which found that concentrations of 0.25–2 mg/mL produced no abnormalities in terms of disturbance of DNA‐related processes, which would indicate strand breakage (clastogenesis) (Kalaivani et al. 2024). Such breakages can lead to deletion, insertion, or rearrangement of chromosome sections, and ultimately to cancer. This lack of genotoxicity was confirmed by an ex vivo test in mice, assessing the effects on erythrocytes, where an increase in micronucleated polychromatic erythrocytes (MNPCEs) indicates clastogenicity or the formation of an abnormal number of chromosomes due to sister chromatid exchange. The incidence of such chromosomal aberrations and micronuclei formation was assessed as the mitotic index (MI) and the percentage of MNPCE, and extracts showed no effects on either, with no differences in cell proliferation compared to vehicle controls (Kalaivani et al. 2024). Furthermore, N. Kumar et al. (2016), using a similar human PBL assay, found that a root extract of AS conferred protection against genotoxicity induced by hydrogen peroxide (N. Kumar et al. 2016). AS leaf extracts offered substantial protection against the mutagenic and genotoxic effects of N‐methyl‐N0‐nitro‐nitroguanidine‐induced chromosomal aberrations in onion root tip cells (Rani et al. 2005). In summary, AS extracts did not induce either chromosome anomalies or micronuclei formation and were able to prevent such occurrences induced by known clastogenic agents.

### 3.4 Organ‐ or System‐Related Toxicity

#### 3.4.1 Cardiotoxicity

Two cases of ventricular tachycardia in elderly men, both of whom recovered, were attributed to AS (Dwivedi et al. 2011). Details of the preparations involved were not available to check for identification, so causality remains unproven, and there is no preclinical evidence to suggest that AS causes cardiac arrhythmias or other heart problems.

#### 3.4.2 Hepatotoxicity

Liver injury, mainly cholestatic in nature, manifested as severe jaundice and pruritus, was reported in 5 patients in Iceland (Björnsson et al. 2020). In Japan, a patient developed cholestatic liver injury with jaundice after using a product labeled as ashwagandha, in combination with multiple anxiolytic drugs (Inagaki et al. 2017). Liver toxicity has not been reported in preclinical tests, except for the study in rats previously cited, which found multiple toxic effects, including centrilobular degeneration of the liver, after feeding with high doses of a crude alkaloidal extract (Arseculeratne et al. 1985). DNA damage by withanone has been suggested as a potential cause of liver toxicity (Siddiqui et al. 2021). Withanone can form non‐labile adducts with deoxynucleosides and with DNA, and thus may interfere with DNA transcription, replication, and repair, resulting in genotoxicity, mutagenesis, and apoptosis. However, withanone can also form adducts with glutathione (GSH), a key molecule involved in drug detoxification, and therefore should only exhibit toxicity at high concentrations, when the protective system involving GSH is overwhelmed (Siddiqui et al. 2021).

Preclinical studies do not explain the observed clinical liver toxicity, which requires further investigation. On the contrary, several studies have found protective effects of withanolides against specific types of liver damage. Vedi et al. (2014) showed that pre‐treatment with AS extracts protected against bromobenzene‐induced damage in Wistar rats, significantly decreasing the levels of liver marker enzymes, TNF‐α, IL‐1β, VEGF, and reduced histopathological damage (Vedi et al. 2014). In vitro studies in rat liver mitochondria suggested this was due to a reduction in oxidative stress. Molecular docking analysis showed involvement of the pro‐inflammatory mediator NF‐κB, and its interaction with withaferin A, withanolide D, and withanolide E (Purushotham et al. 2017). Azab et al. (2022) found that 470 mg of AS root extract (containing 1.5% withanolides) ameliorated γ‐radiation damage in Sprague–Dawley rats, confirmed by histopathological investigation of spleen and liver tissues; the authors attributed this protection to its antioxidants and anti‐inflammatory effects (Azab et al. 2022). Khalil et al. (2023) investigated the effects of AS‐loaded nanocapsules in a thioacetamide‐induced hepatic encephalopathy Wistar rat model and found that they downregulated hepatic NF‐κB expression, maintained hepatic and brain architecture, and improved behavioral changes (Khalil et al. 2023).

Patel et al. (2019) explored the role of withaferin‐A in treating nonalcoholic steatohepatitis using the methionine‐choline‐deficient diet and high‐fat diet C57BL/6N mouse models and found that it prevented and ameliorated liver injury in both models, shown by lower serum aminotransaminases and reduced hepatic steatosis, liver inflammation, and fibrosis; furthermore, they demonstrated a leptin‐independent hepatoprotective effect in the high‐fat diet group (D. P. Patel et al. 2019). Xia et al. (2022) have reviewed the potential of withaferin‐A as a treatment for acute liver injury, nonalcoholic fatty liver disease, liver fibrosis, and especially for liver cancer. Withaferin‐A is known to protect against liver cancer both in rodent and in vitro models, and based on experimental evidence and the signaling pathways involved, withaferin‐A selectively kills liver tumor cells compared to normal hepatocytes (Xia et al. 2022). Withaferin A inhibits liver cancer tumorigenesis in HEp‐2 cells by blocking aerobic glycolysis (Zhou et al. 2024).

#### 3.4.3 Endocrine Toxicity

Rare cases of thyrotoxicosis following AS administration have been reported (Hayashi et al. 2024; Kamal et al. 2022; Ziganshina and Joseph 2022). Orally administered root extract (1.4 g/kg daily) to Swiss mice for 20 days increased serum levels of 3,3′,5‐triiodothyronine, tetraiodothyronine levels, and thyroxine, and hepatic glucose‐6‐phosphatase activity (Panda and Kar 1998, 1999). Similar findings were reported from a study in hypothyroid male albino rats induced by propylthiouracil (Abdel‐Wahhab et al. 2019). Verma et al. (2021) conducted a placebo‐controlled trial in 80 healthy volunteers and found that 300 mg of an extract of AS administered twice daily had no effect on thyroid profiles. However, in subclinical hypothyroid patients, Sharma et al. (2018) showed that treatment with AS may be beneficial for normalizing thyroid indices. Thus, although preclinical evidence suggests that ashwagandha root extract may stimulate thyroid activity, this is reversible, often asymptomatic, and may in some cases be beneficial. The topic will be assessed in more detail in a separate publication (Vollmer and Brendler 2026).

## 4 Conclusion

The great majority of safety tests on AS, when prepared according to traditional principles (i.e., extracted with water or hydroalcoholic solvents) show that it is an unusually safe herb. There is no preclinical evidence to support the suggestion that AS is unsafe in pregnancy, and no verifiable clinical evidence to suggest it is harmful to either mother or offspring. Occasionally, ethnobotanical surveys cite specific toxic effects of AS related to “abortion” and “sterility” (Al‐Qura'n 2005), but these are opinions held by respondents in a particular population or region, without substantiating evidence being provided (Brendler 2025).

AS is widely used for the treatment and prevention of inflammatory, degenerative, and neuropsychiatric diseases in the elderly, infirm, and seriously ill, without reported problems. It shows promise in cancer prevention and treatment and is the subject of intensive research for this purpose (Zhang et al. 2024). The use of AS in adjunctive therapy to increase efficacy and reduce toxicity of radio‐ and chemotherapy appears to be justified, and these aspects and mechanisms have been comprehensively reviewed (Bashir et al. 2023; Mikulska et al. 2023). For example, a purified extract, standardized to 1.5% withanolides, protected against doxorubicin‐induced cardiac toxicity in Wistar rats (Hamza et al. 2008), and a further study demonstrated that withaferin A is cardioprotective (in vivo and in vitro) at low doses and prevents myocardial ischemia injury in AMPK‐DN transgenic mice (Guo et al. 2019). Ovarian cancer induces cardiac cachexia, leading to loss of cardiac function, and a study in female mice found that withaferin A rescued the heart weight and improved systolic and diastolic dysfunction in tumor‐bearing NSG mice (Kelm et al. 2020).

AS‐based dietary supplements are traditionally prepared from the root (D. Langade et al. 2019; Mikulska et al. 2023), and more recently also from leaves. In terms of safety concerns, it is noteworthy, however, that P. Kumar et al. (2023) stress that “irrespective of the root or leaf, the toxic effects are realized only at concentrations beyond the prescribed safe limit of the extract, which is 500–2000 mg/kg body weight per day” (P. Kumar et al. 2023). AS has an almost unparallelled record of safety, even in vulnerable patient groups, where it has specific applications. However, further clarification is needed on the effects of individual constituents, doses and patient‐related conditions which may account for the reported clinical liver toxicity.

## Author Contributions

**Elizabeth M. Williamson:** conceptualization, data curation, formal analysis, investigation, methodology, resources, writing – original draft, writing – review and editing. **Thomas Brendler:** conceptualization, investigation, project administration, resources, writing – original draft, writing – review and editing.

## Conflicts of Interest

The authors declare no conflicts of interest.

## Data Availability

The data that support the findings of this study are available from the corresponding author upon reasonable request.
